# Vitamin E improves antioxidant status but not lipid metabolism in laying hens fed a aged corn-containing diet

**DOI:** 10.5713/ajas.19.0934

**Published:** 2020-04-12

**Authors:** X. M. Ding, Y. D. Mu, K. Y. Zhang, J. P. Wang, S. P. Bai, Q. F. Zeng, H. W. Peng

**Affiliations:** 1 Institute of Animal Nutrition, Sichuan Agricultural University, Key laboratory of Animal Disease-resistant Nutrition, Ministry of Education, Key laboratory of Animal Disease-resistant Nutrition, Sichuan Province Chengdu, 611130, China

**Keywords:** Vitamin E, Aged Corn, Lipid Metabolism, Antioxidant, Laying Hens

## Abstract

**Objective:**

The objective of this study was to determine whether a dietary vitamin E (VE) supplement could alleviate any detrimental effects of aged corn on lipid metabolism and antioxidant status in laying hens.

**Methods:**

The experiment consisted of a 2×3 factorial design with two corn types (normal corn and aged corn (stored for 4 yr) and three concentrations of VE (0, 20, and 100 IU/kg). A total of 216 Lohmann laying hens (50 wk of age) were randomly allocated into six treatment diets for 12 wk. Each treatment had 6 replicates of 6 hens per replicate.

**Results:**

The results show that aged corn significantly decreased the content of low-density lipoprotein cholesterol (p<0.05), and reduced chemokine-like receptor 1 (CMKLR1) mRNA expression (p<0.05) in the liver compared to controls. Diet with VE did not alter the content of crude fat and cholesterol (p>0.05), or acetyl-CoA carboxylase, lipoprotein lipase, fatty acid synthase or CMKLR1 mRNA expression (p>0.05) in the liver among treatment groups. Aged corn significantly increased the content of malondialdehyde (MDA) (p<0.05) and decreased superoxide dismutase (SOD) activity (p<0.05) in the liver. The VE increased the content of MDA (p<0.05) but decreased glutathione peroxidase (GSH-Px) activity in serum (p<0.01) and in the ovaries (p<0.05). Adding VE at 20 and 100 IU/kg significantly increased GSH-Px activity (p<0.05) in liver and in serum (p<0.01), 100 IU/kg VE significantly increased SOD activity (p<0.05) in serum. Aged corn had no significant effects on *GSH-Px* mRNA or *SOD* mRNA expression (p<0.01) in the liver and ovaries. Addition of 100 IU/kg VE could significantly increase *SOD* mRNA expression (p<0.01) in the liver and ovary.

**Conclusion:**

Aged corn affected lipid metabolism and decreased the antioxidant function of laying hens. Dietary VE supplementation was unable to counteract the negative effects of aged corn on lipid metabolism. However, addition of 100 IU/kg VE prevented aged corn-induced lipid peroxidation in the organs of laying hens.

## INTRODUCTION

Research on the impacts of aged corn in the feed of production animals, such as laying hens, is increasing in importance due to long term storage in countries for the eventuality of a natural disaster. A considerable amount of these stored grains can be lost due to interactions among various physical, chemical, and biological factors associated with storage conditions [1]. Previous studies have shown that the quality and nutrient content of corn decreased with increased storage time [2]. A significant decrease in pH, digestible lysine content, and thiamine content, as well as an increase in titratable acidity were observed when corn was stored for an extended period [3]. Further, corn lipids like fatty acids are susceptible to oxidation and rancidity [4]. Free fatty acid can be easily oxidized to produce H_2_O_2_, and thus affect catalase (CAT) and peroxidase (POD) activities in corn [5].

Feeding animals corn that has been stored long-term can cause adverse effects. Feeding aged corn resulted in reduced antioxidant function in broilers [6]. Ducks fed aged corn were more likely to have oxidative damage that reduced growth performance [7]. However, the effects of aged corn on lipid metabolism and antioxidant systems in laying hens remain largely unknown.

Vitamin E (VE) is a necessary fat-soluble vitamin for poultry. As a natural antioxidant, it plays an important role in scavenging free radicals, preventing lipids peroxidation, and protecting animals from the adverse effects caused by oxidative stress [8]. The VE improved antioxidant function of meat ducks fed aged corn diets [9]. Dietary supplementation with 200 mg/kg VE enhanced the antioxidant capacity of laying hens [10]. A previous study showed that VE could not prevent the visible yellow coloration of backfat and lipofuscin in pigs fed high oxidant diets, although it did decrease the extractable lipid content [11]. Our study aimed to determine if dietary supplementation with VE to hens fed aged corn could influence on lipid metabolism and antioxidant status.

## MATERIALS AND METHODS

### Aged corn

The aged corn used in this study was previously stored for 4 yr and was obtained from the national barns in Jilin, China. The control corn used in this study was stored for half a year and was obtained from Ningxia, China. All corn samples were stored in brick structures. Phytochemical properties of corn (Table 1) were the same as was previously published by Mu et al [12].

### Experimental birds, diets, and management

The present study was performed on a poultry farm (Ya’an, China), and the Animal Care and Use Committee of Sichuan Agricultural University approved all experimental procedures. A total of 216 Lohmann laying hens (50 wk of age) were randomly divided into 6 treatment groups that consisted of 6 replicates for each treatment in a completely randomized design with a 2×3 factorial arrangement of treatments (Corn× VE), 2 cages per replicates, 3 birds per cage (38.1 cm-width× 50 length×40 height). Hens were given diets supplemented with one of two corn types (normal corn or aged corn) and 3 concentrations of VE (dl-α-tocopheryl acetate, 0, 20, 100 IU/kg). Each treatment was uniformly distributed in the laying house to minimize environmental effects. All hens were housed in stainless steel cages and the room environment was controlled at 22°C and had a daily lighting schedule of 16 h light and 8 h dark. Hens were allowed *ad libitum* access to experimental diets and water throughout the 12 wk experiment. The basal diet (Table 2) was based on corn and soybean meal, with composition and nutrient formulation in line with the Agricultural Trade Standardization of China (NY/T33-2004). All diets were provided in mash form.

### Assay of antioxidant enzymes in serum, liver, and ovaries

At the end of the 12 wk experimental period, 6 birds from each treatment were randomly selected (one bird per replicate). Blood (10 mL) was drawn from the wing vein and placed in tubes to allow serum collection. Feed was not withdrawn from the trough before the blood was collected. After the blood was collected, the tubes containing blood were centrifuged for 10 min (3,000×g) at 4°C, and then serum was collected in new tubes and stored at −20°C until further analyses of enzyme activity. After blood collection, birds were euthanized by cervical dislocation. Liver and ovary samples were obtained, and a section of each sample was frozen in liquid nitrogen, then stored at −80°C until analyzed for enzyme activity and gene expression. Liver and ovarian samples were homogenized (PowerGen 125, Fisher Scientific, Pittsburgh, PA, USA) for subsequent antioxidant status determination. Analyses included enzymatic activity of superoxide dismutase (SOD) and glutathione peroxidase (GSH-Px) for total antioxidant capacity (T-AOC) and content of malondialdehyde (MDA) using the same procedures as described by instructions with assay kits obtained from Nanjing Jiancheng Bioengineering Institute (Nanjing, Jiangsu, China). Triplicate analyses were performed, and the mean was used for each sample.

### Determination of liver lipid metabolism parameters

Another section of each liver sample was stored at −20°C for the determination of crude fat and cholesterol concentrations. The specific assay kits were purchased from the Nanjing Jiancheng Bioengineering Institute (Nanjing, China). Other liver samples, which were stored at −80°C, were analyzed for lipid metabolism parameters and gene expression by means of RNA extraction and measurement.

### mRNA expression in liver and/or ovaries

Total RNA was isolated from frozen livers using Trizol reagent (catalogue No.9109, Takara, Otsu, Japan) according to the manufacturer’s instructions. The ratio of absorbance at 260 nm to at 280 nm as well as the banding pattern on a 1% agarose gel were used to verify the purity and quality of RNA samples (Beckman DU-800; Beckman Coulter, Inc., Fullerton, CA, USA). The RNA concentration was determined by measuring absorbance at 260 nm. After extraction of total RNA, followed by reverse transcription (RT) inactivation at 85°C for 5 s using PrimeScriptTM RT reagent kit (catalogue No. RR047A, Takara, Japan). A 1-μL portion of the RT products was used directly for real-time polymerase chain reaction (PCR). Real-time PCR was performed on an ABI-7900 instrument (Advanced Biosystems, Foster, CA, USA). Oligonucleotide primers were used to detect the gene expression of β-actin, fatty acid synthase (*FAS*), acetyl-CoA carboxylase (*ACC*), lipoprotein lipase (*LPL*), chemokine-like receptor 1 (*CMKLR1*), *GSH-Px*, and *SOD1* using the SYBR Green system (catalog No. RR820A, Takara, Japan). The sequences of the primers and length of the products are given in Table 3. The reaction mixture (10 μL) contained 5 μL of fresh SYBR Premix Ex TaqII (Tli RNaseH Plus, Takara, Japan) and 0.2 μL ROX Reference Dye II (50×), 0.8 μL of the primers, 1 μL of RT products and 3 μL dH_2_O. The following PCR protocol was used with the ABI-7900: 1 cycle (at 95°C for 30 s), 40 cycles (at 95°C for 5 s and 60°C for 31 s) and 1 cycle (at 95°C for 15 s, at 60°C for 1 min and at 95°C for 15 s). The standard curve of each gene was run triplicate for obtaining reliable amplification efficiency values. At the end of amplification, melting curve analysis was performed to identify amplification specificity. β-Actin transcript was used to standardize the results by eliminating variations in mRNA and cDNA quantity and quality, and each mRNA level was expressed as its ratio to β-actin mRNA. The relative quantification of gene expression among the treatment groups was analyzed by the 2^−ΔΔCT^ method [13].

### Statistical analyses

The Glimmix procedure of SAS 9.2 (SAS Institute Inc, Cary, NC, USA) was used to test the effects of supplemental VE in normal corn and aged corn diets. Data were analyzed as a 2×3 (Corn×VE) factorial arrangement of treatments by 2-way analysis of variance with a model that included the main effects of corn and VE, as well as their interaction. When an effect was significant, means were compared by Duncan’s multiple comparison tests to determine specific differences between means. Statistical significance was assigned at p< 0.05.

## RESULTS

### Hepatic lipid metabolism and gene expression

Neither aged corn nor dietary VE had any significant influence on hepatic lipid metabolism of laying hens (p>0.05; Table 4), except that the content of low-density lipoprotein cholesterol (LDL-C) in hens fed the aged corn diet was significantly decreased (p<0.05) compared to normal corn. The relative expression of *CMKLR1* in hens fed the aged corn diet was also significantly decreased (p<0.05; Table 5) compared to normal corn. Gene expression of *ACC*, *LPL*, and *FAS* showed no significant difference among groups (p>0.05).

### Antioxidant status in serum

In hens fed the aged corn diets, the serum content of MDA was increased (p<0.05) and the activity of GSH-Px was decreased (p<0.01). The activity of SOD was increased with dietary VE addition; with the 100 IU/kg VE group being significantly higher than the 0 IU/kg VE group (p<0.05). The activity of GSH-Px was increased (p<0.01) in the 20 IU/kg and 100 IU/kg VE group compared to 0 IU/kg VE group (Table 6).

### Antioxidant status in liver

Compared to the normal corn group, the content of hepatic MDA was increased (p<0.05) by feeding the aged corn diet, and the activity of SOD was decreased (p<0.05) by feeding the aged corn diet. Dietary VE had no effect on the hepatic activity of SOD or T-AOC, or on the content of MDA. However, the activity of GSH-Px significantly increased (p<0.05) with the VE addition in diets (Table 7).

### Antioxidant status in ovary

The activity of SOD was decreased (p<0.05) in the aged corn diets compared to the normal corn diets (Table 8). The content of MDA was decreased and the activity of SOD, GSH-Px, and T-AOC did not show significant differences among the groups (p>0.05).

### Antioxidant enzyme gene expressions

As presented in Table 9, expression of *SOD1* mRNA in the ovaries was significantly upregulated by feeding of aged corn (p<0.01). *GSH-Px* and *SOD1* mRNA expression in liver and *GSH-Px* mRNA expression in the ovaries showed no significant difference among groups fed with normal corn diet and the aged corn diet (p>0.05). Compared to 0 and 20 IU/kg VE diet groups, expression of *SOD1* mRNA in the liver and ovaries were significantly increased when birds were fed diets containing 100 IU/kg VE. The observed interaction between corn and VE (p<0.05) was that *GSH-Px* and *SOD1* mRNA expression in the ovaries was higher in aged corn diet containing 100 IU of VE/kg versus those fed aged corn at lower VE supplementation.

## DISCUSSION

Previous studies have shown changes in the chemical composition and nutritive value of cereal grains during storage [14]. The fatty acid value and POD activity are important parameters for the quality evaluation of corn in storage. In our study, the content of MDA and the acidity of fatty acids in aged corn were higher than the normal corn, while the CAT and POD in aging corn were lower than the normal corn. The activity of CAT and POD in corn gradually decreased with increased storage time [15]. Meanwhile, the fatty acid (FA) profile in aged corn was different than normal corn. The content of mycotoxins was small, so the effects of aged corn on hens in this experiment were caused by changes in the nutritional components of corn, such as oxidation of fatty acids.

The cholesterol content of the yolk is primarily dependent on the cholesterol content of triglyceride-rich lipoproteins-very low-density lipoprotein (VLDL) and vitellogenin. Almost 95% cholesterol is derived from VLDL made in the liver, where it is synthesized and transported in the plasma to the ovaries where they are taken up into the developing follicles by receptor mediated endocytosis [16]. When lipid peroxidation products present in oxidized oils were fed to rats and laying hens, it caused a reduction in the concentration of triacylglycerols and cholesterol in the liver and plasma [17]. In the present study, no differences in liver total cholesterol (TC) were observed. This result was in accordance with a previous study, which showed no differences in the level of hepatic TC in laying hens fed oxidized oil [18]. Serum TC was not significantly affected by oxidized sunflower oil in rats [13]. However, aged corn significantly decreased the content of LDL-C in the liver. Oxidized sunflower oil significantly decreased liver TC, high-density lipoprotein cholesterol (HDL-C) and VLDL-cholesterol (VLDL-C) content in rats [15]. In our previous study, aged corn significantly decreased the cholesterol content including TC, LDL-C, and VLDL-C in serum [13]. Dietary VE supplementation in lacaune ewes decreased serum LDL-C content [19] which agrees with [20] who found VE decreased the content of TC, total triglyceride (TG), HDL-C, and VLDL-C in serum of rats. In this study, VE supplementation did not affect the lipid metabolism in laying hens. In our study, the aged corn was less oxidized than the oxidized soybean oil [18] and sunflower oils used in other prior studies [21,22]; therefore, the decrease in crude fat and TC was not significant.

CMKLR1, a G protein-coupled receptor, and its ligand chemerin are known to be involved in adipogenesis [23]. CMKLR1 is expressed in the liver [24]. In human and rodent fatty liver, and in fibrotic liver of mice fed a methionine–choline deficient diet, CMKLR1 is reduced [25]. Lipid loading of hepatocytes does not affect CMKLR1 levels, similar to adipocytes where enhanced triglyceride accumulation has no effect on this receptor [26]. In the present study, *CMKLR1* mRNA expression was significantly decreased in the hens fed the aged corn.

The intake of exogenous free radicals can cause a decrease in antioxidants. The storage of corn resulted in many free radicals, and laying hens fed aged corn had a lower serum antioxidant status [6]. The hens fed aged corn were more susceptible to oxidative damage and decreased laying performance [7]. Oxidative stress induced by lipid peroxidation products has been proposed as a possible mechanism involved in the toxicity of thermally oxidized oil [27]. Exposure to oxidized sunflower oil increased plasma MDA, hepatic reactive oxygen species and carbonyl group concentrations [28]. The antioxidant enzymes SOD and GSH-Px are the main elements of the first level of antioxidant defense in the cell because they form a major protective system against oxidative damage. In the present study, serum and liver MDA were significantly increased, the serum GSH-Px, liver and ovary SOD were significantly decreased in hens fed the aged corn. These results suggest that enhanced peroxidation leads to an imbalance of the pro-oxidant/antioxidant system. The VE is a potent chain breaking antioxidant, and its primary role is to prevent lipids peroxidative damage in the tissue [29]. In our study, dietary VE supplementation elevated the activities of SOD in serum and GSH-Px both in serum and liver of laying hens. Our results are consistent with a previous study, which showed VE improved the activities of total SOD (T-SOD) and GSH-Px in plasma and liver of laying hens fed with the oxidized sunflower oil diet [28]. Our results did not show significant effects of aged corn and VE on ovarian antioxidant parameters. However, the relative expression of ovarian *SOD1* mRNA was increased in hens fed the aged corn diets. This is inconsistent with the activity of SOD being decreased in ovaries of layers fed with the aged corn diet, which may be relevant to how much of the *SOD1* mRNA was translated. This indicates that aged corn (or metabolites) may regulate the expression of antioxidant enzyme genes at the level of transcription and translation. In general, these results showed that feeding aged corn leads to an imbalance of the pro-oxidant/antioxidant system, and VE supplementation efficiently reversed this imbalance.

## CONCLUSION

In conclusion, feeding aged corn affected lipid metabolism and decreased the antioxidant function of laying hens. Dietary supplementation of VE was unable to counteract the negative effects of aged corn on lipid metabolism in laying hens. However, addition of 100 IU/kg VE prevented aged corn-induced lipid peroxidation in laying hens, possibly via a direct increase in antioxidant enzyme activities and enhancing the relative expressing of antioxidant genes. Future studies will be performed and will hopefully confirm or reproduce the results of the current study.

## Figures and Tables

**Table 1:** Phytochemical properties of aged corn (stored for 4 yr) and normal corn (air-dried basis)

Items	Content	

	Aged corn	Normal corn
Moisture (%)	13.06	14.31
Crude protein (%)	7.73	7.56
Gross energy (cal/g)	3,846	3,799
Crude fat (%)	3.31	3.42
Acidity of fatty acids (KOH mg/100 g)	126	64
MDA (nmol/mL)	96.03	40.30
CAT (U/mg)	17.00	28.49
POD (U/mg)	34.26	64.93
Aflatoxin (μg/kg)	-	1.9
Zealerenol (μg/kg)	87.4	63.4
Deoxynivalenol (μg/kg)	240.9	-
Fatty acid methyl esters (mg/g)		
Palmitic (C16:0)	2.45	3.16
Stearic (C18:0)	0.26	0.31
Oleic (C18:1)	3.87	5.73
Linoleic (C18:2)	9.23	11.39
Linolenic (C18:3)	0.01	0.32

“-” represents below limit of detection.

MDA, malondialdehyde; CAT, catalase; POD, peroxidase.

**Table 2:** Dietary composition, and formulated energy and nutrient content of the basal diet (air-dried basis, %)

Item (%)	Amount
Corn1)	57.05
Soybean meal, 43%CP	22.40
Wheat bran	7.81
Soybean oil	2.00
L-lysine	0.13
DL-methionine	0.11
Calcium carbonate	8.35
Calcium hydrophosphate	1.12
NaCl	0.40
Choline Chloride	0.10
Vitamin premix2)	0.03
Mineral premix3)	0.50
Calculated nutrient and energy content	
ME (MJ/kg)	11.10
CP	15.50
Ca	3.51
Total P	0.57
Available phosphorus	0.32
Lysine	0.79
Methionine	0.32
Vitamin E4)	8.10 (3.13)

CP, crude protein; ME, metabolizable energy.

1) Corn utilized was supplied entirely from normal or aged in experimental diets.

2) Provided per kilogram of diet: vitamin A, 8,000 IU; vitamin D_3_, 1,600 IU; vitamin K, 0.5 mg; vitamin B_1_, 0.8 mg; vitamin B_2_, 2.5 mg; vitamin B_6_, 3.0 mg; vitamin B_12_, 0.004 mg; folic acid, 0.25 mg; niacin, 20 mg; Ca-pantothenate acid, 2.2 mg; biotin, 0.10 mg; vitamin E, according to the amount of each treatment (dl-α-tocopheryl acetate, 0, 20, or 100 mg).

3) Provided per kg of diet: 60 mg Mn (as MnSO_4_); 80 mg Zn (as ZnSO_4_); 8 mg Cu (as CuSO_4_·5H_2_O); 60 mg Fe (as FeSO_4_·7H_2_O); 0.35 mg I (as KI); and 0.30 mg Se (as Na_2_SeO_3_·5H_2_O).

4) VE content in normal corn and aged corn basal diet was 8.10 and 3.13 IU/kg respectively.

**Table 3:** Primers sequence used for the determination of lipid metabolism and antioxidant function of laying hens

Item	Primer sequence	Primer length
β-actin	F primer: TGCGTGACATCAAGGAGAAG	152
	R primer: TGCCAGGGTACATTGTGGTA	
*ACC*	F primer: CCTGGTCAACGTGATGAATG	152
	R primer: AGTTCCAGCAGAGGCAAAGA	
*LPL*	F primer: TGCTGGTCCCACCTTTGAGTA	185
	R primer: TGCAGGACATCCACAAAGTCA	
*FAS*	F primer: ACTGTGGGCTCCAAATCTTCA	164
	R primer: CAAGGAGCCATCGTGTAAAGC	
*CMKLR1*	F primer: CGGTCAACGCCATTTGGT	65
	R primer: GGGTAGGAAGATGTTGAAGAGGAA	
*GSH-Px*	F primer: ATCCGCTTCCACGACTTCC	137
	R primer: ATCTTCACGTTGCGTTTGCT	
*SOD1*	F primer: TTGTCTGATGGAGATCATGGCTTC	98
	R primer: TGCTTGCCTTCAGGATTAAAGTGAG	

*ACC*, acetyl-CoA carboxylase; *LPL*, lipoprotein lipase; *FAS*, fatty acid synthase; *CMKLR1*, chemokine-like receptor 1; *GSH-Px*, glutathione peroxidase; *SOD1*, superoxide dismutase 1.

**Table 4:** Effects of vitamin E supplementation on liver lipid metabolism of laying hens fed diets containing aged (4 yr) or normal corn

Corn	Vitamin E (IU/kg)	CF (%)	TC (mmol/g)	HDL-C (mmol/g)	LDL-C (mmol/g)	VLDL-C (μmol/g)
Normal corn	0	21.54	0.84	0.70	0.08	1.50
	20	21.15	0.81	0.41	0.05	1.53
	100	19.06	0.80	0.35	0.10	1.47
Aged corn	0	19.32	0.77	0.29	0.03	1.72
	20	18.91	0.81	0.43	0.03	1.46
	100	18.38	0.87	0.34	0.02	1.64
SEM		1.68	0.05	0.11	0.02	0.17
Main effect						
Corn	Normal corn	20.58	0.82	0.49	0.07^a^	1.50
	Aged corn	18.87	0.82	0.35	0.03^b^	1.61
Vitamin E (IU/kg)	0	20.43	0.81	0.50	0.05	1.61
	20	20.03	0.81	0.42	0.04	1.50
	100	18.72	0.83	0.34	0.06	1.56
p-value	Corn	0.22	0.95	0.14	0.03	0.45
	Vitamin E	0.57	0.82	0.39	0.66	0.81
	Corn×vitamin E	0.87	0.29	0.11	0.57	0.67

Each mean represents 6 layers.

CF, crude fat; TC, total cholesterol; HDL-C, high-density lipoprotein cholesterol; LDL-C, low-density lipoprotein cholesterol; VLDL-C, very low-density lipoprotein cholesterol; SEM, standard error of the mean.

a,b Means with different superscripts within a column differ significantly (p<0.05), and comparisons only conducted within each main effect.

**Table 5:** Effects of vitamin E supplementation on the relative expression of liver lipid metabolism gene of laying hens fed diets containing aged corn

Corn	Vitamin E (IU/kg)	*ACC*	*LPL*	*FAS*	*CMKLR1*
Normal corn	0	1.12	1.04	1.42	1.12
	20	1.40	1.54	1.72	1.43
	100	0.99	2.85	0.98	1.90
Aged corn	0	1.62	1.35	1.78	0.78
	20	1.66	1.33	1.24	0.81
	100	1.05	1.54	1.83	1.05
SEM		0.34	0.44	0.30	0.28
Main effect					
Corn	Normal corn	1.17	1.81	1.37	1.48^a^
	Aged corn	1.45	1.41	1.62	0.88^b^
Vitamin E (IU/kg)	0	1.37	1.20	1.60	0.95
	20	1.53	1.44	1.48	1.12
	100	1.02	2.19	1.40	1.47
p-value	Corn	0.33	0.26	0.32	0.01
	Vitamin E	0.31	0.07	0.80	0.17
	Corn×vitamin E	0.81	0.18	0.09	0.65

Each mean represents 6 laying hens.

*ACC*, acetyl-CoA carboxylase; *LPL*, lipoprotein lipase; *FAS*, fatty acid synthase; *CMKLR1*, chemokine-like receptor 1; SEM, standard error of the mean.

a,b Means with different superscripts within a column differ significantly (p<0.05), and comparisons only conducted within each main effect.

**Table 6:** Effects of vitamin E supplementation on serum antioxidant status of laying hens fed diets containing aged (4 yr) or normal corn

Corn	Vitamin E (IU/kg)	MDA (nmol/mg)	SOD (U/mg)	GSH-Px (U/mg)	T-AOC (U/mg)
Normal corn	0	5.73	4.67	1,109.05	3.45
	20	3.89	5.17	1,404.28	4.42
	100	4.32	7.17	1,671.91	4.09
Aged corn	0	6.05	5.17	980.48	3.98
	20	5.73	5.25	1,102.86	3.29
	100	5.16	6.08	1,137.62	4.07
SEM		0.58	0.71	83.49	0.54
Main effect					
Corn	Normal corn	4.64^b^	5.66	1,395.08^a^	3.99
	Aged corn	5.65^a^	5.50	1,073.65^b^	3.78
Vitamin E (IU/kg)	0	5.89	4.92^b^	1,044.76^b^	3.72
	20	4.81	5.21^ab^	1,253.57^a^	3.85
	100	4.74	6.63^a^	1,404.76^a^	4.08
p-value	Corn	0.04	0.77	<0.01	0.64
	Vitamin E	0.10	0.04	<0.01	0.79
	Corn×vitamin E	0.42	0.52	0.07	0.31

Each mean represents 6 laying hens.

MDA, malondialdehyde; SOD, superoxide dismutase; GSH-Px, glutathinone peroxidase; T-AOC, total antioxidant capacity; SEM, standard error of the mean.

a,b Means with different superscripts within a column differ significantly (p<0.05), and comparisons only conducted within each main effect.

**Table 7:** Effects of vitamin E supplementation on liver antioxidant status of laying hens fed diets containing aged (4 yr) or normal corn

Corn	Vitamin E (IU/kg)	MDA (nmol/mg)	SOD (U/mg)	GSH-Px (U/mg)	T-AOC (U/mg)
Normal corn	0	1.93	8.78	171.41	1.13
	20	0.86	9.43	241.73	1.36
	100	1.03	9.74	232.13	1.08
Aged corn	0	1.96	7.34	110.89	0.70
	20	1.86	8.07	240.44	0.95
	100	1.76	8.61	276.19	1.16
SEM		0.32	0.59	39.00	0.20
Main effect					
Corn	Normal corn	1.27^b^	9.32^a^	215.09	1.19
	Aged corn	1.86^a^	8.01^b^	209.17	0.94
Vitamin E (IU/kg)	0	1.94	8.06	141.15^b^	0.91
	20	1.36	8.75	241.09^a^	1.16
	100	1.39	9.17	254.16^a^	1.12
p-value	Corn	0.03	0.01	0.85	0.13
	Vitamin E	0.13	0.18	0.01	0.43
	Corn×vitamin E	0.30	0.97	0.42	0.36

Each mean represents 6 laying hens.

MDA, malondialdehyde; SOD, superoxide dismutase; GSH-Px, glutathinone peroxidase; T-AOC, total antioxidant capacity; SEM, standard error of the mean.

a,b Means with different superscripts within a column differ significantly (p<0.05), and comparisons only conducted within each main effect.

**Table 8:** Effects of vitamin E supplementation on ovary antioxidant status of laying hens fed diets containing aged (4 yr) or normal corn

Corn	Vitamin E (IU/kg)	MDA (nmol/mg)	SOD (U/mg)	GSH-Px (U/mg)	T-AOC (U/mg)
Normal corn	0	0.17	8.34	1,029.39	0.51
	20	0.12	9.49	1,365.82	0.62
	100	0.07	9.48	1,063.40	0.67
Aged corn	0	0.16	7.48	739.78	0.47
	20	0.15	7.11	1,199.04	0.49
	100	0.15	8.95	1,146.48	0.51
SEM		0.03	0.63	396.42	0.12
Main effect					
Corn	Normal corn	0.12	9.10^a^	1,152.87	0.60
	Aged corn	0.15	7.84^b^	1,028.43	0.49
Vitamin E (IU/kg)	0	0.16	7.91	884.58	0.49
	20	0.13	8.30	1,282.43	0.56
	100	0.11	9.21	1,104.94	0.59
p-value	Corn	0.13	0.02	0.70	0.27
	Vitamin E	0.22	0.12	0.61	0.72
	Corn×vitamin E	0.24	0.31	0.89	0.86

Each mean represents 6 laying hens.

MDA, malondialdehyde; SOD, superoxide dismutase; GSH-Px, glutathinone peroxidase; T-AOC, total antioxidant capacity; SEM, standard error of the mean.

a,b Means with different superscripts within a column differ significantly (p<0.05), and comparisons only conducted within each main effect.

**Table 9:** Effects of vitamin E supplementation on the relative expression of liver and ovary antioxidant gene of laying hens fed diets containing aged (4 yr) or normal corn

Corn	Vitamin E (IU/kg)	Liver		Ovary	
	
		GSH-Px (U/mg)	SOD1 (U/mg)	GSH-Px (U/mg)	SOD1 (U/mg)
Normal corn	0	1.25	1.44	0.92^b^	0.83^b^
	20	1.69	2.22	3.15^a^	1.49^b^
	100	1.82	3.60	2.63^ab^	1.72^b^
Aged corn	0	1.46	2.39	3.36^a^	2.45^b^
	20	1.42	1.72	2.29^ab^	1.65^b^
	100	1.54	2.67	3.48^a^	5.14^a^
SEM		0.31	0.42	0.58	0.62
Main effect					
Corn	Normal corn	1.59	2.42	2.23	1.34^b^
	Aged corn	1.48	2.26	3.05	3.08^a^
Vitamin E (IU/kg)	0	1.35	1.91^b^	2.14	1.64^b^
	20	1.56	1.97^b^	2.72	1.57^b^
	100	1.68	3.14^a^	3.05	3.43^a^
p-value	Corn	0.66	0.64	0.10	<0.01
	Vitamin E	0.57	<0.01	0.29	<0.01
	Corn×vitamin E	0.65	0.08	0.03	0.04

Each mean represents 6 laying hens.

*GSH-Px*, glutathione peroxidase; *SOD1*, superoxide dismutase 1; SEM, standard error of the mean.

a,b Means with different superscripts within a column differ significantly (p<0.05), and comparisons only conducted within each main effect.
